# Natural product derived phytochemicals as modulators of cancer stemness and drug resistance

**DOI:** 10.3389/fonc.2026.1940814

**Published:** 2026-09-07

**Authors:** Asif Khurshid Qazi, Muhammad Omais Parray, Sajad Hussain Syed

**Affiliations:** 1 Cancer Pharmacology Division, CSIR-Indian Institute of Integrative Medicine, Branch Laboratory Sanat Nagar, Srinagar, India; 2 Academy of Scientific and Innovative Research (AcSIR), Ghaziabad, India

**Keywords:** cancer, essential oil, multidrug resistance, stem cells, tumour

## Abstract

Natural secondary metabolites, especially Essential oils (EOs), are compounds made by aromatic plants. These oils contain a variety of bioactive molecules with distinct effects on the body and are known for their strong, low toxicity and wide-ranging biological activities. These herbal concentrates have antimicrobial, antioxidant, anti-inflammatory, antiproliferative, and anticancer effects, mainly due to their terpene and phenolic compounds. Research shows that volatile oils can target cancer cells and may boost the effectiveness of conventional chemotherapy. Using plant based compounds to target drug resistant cancer stem cells (CSCs) is a newer strategy in cancer prevention and treatment. Even though we know more about how cells turn cancerous, cancer is still a major global health issue. This is mostly because cancer cells can resist many different standard drugs. Tumours turn resistant due to their ability to adapt and their frequent exposure to anti-cancer drugs. This review summarises current preclinical evidence on the potential of EOs to modulate CSC associated signalling pathways and overcome CSC mediated drug resistance. It highlights the multitarget mechanisms by which EOs influence CSC maintenance, self-renewal and therapeutic resistance, whilst critically acknowledging that the available evidence is largely limited to preclinical studies. It also emphasises the need for standardised EO characterisation, detailed mechanistic investigations, advanced drug delivery systems to improve bioavailability and pharmacokinetics and well-designed clinical studies to establish the safety, efficacy and therapeutic value of EOs in precision oncology.

## 1 Introduction

Cancer is still one of the leading causes of death worldwide, even though there have been advances in chemotherapy, radiation therapy, immunotherapy and targeted treatments (1). A major challenge in effective cancer treatment is drug resistance and the difficulty in identifying cancer stem cells (CSCs) (2). CSCs are a group of tumour cells known for their ability to self-renew, differentiate, start new tumours, spread to other areas and resist standard treatments. These cells contribute significantly to tumour recurrence and poor prognosis (3).

Natural products have attracted significant attention as substitute anti-cancer agents (4). Amongst them, Essential Oils (EOs), complex mixtures of volatile phytochemicals extracted from aromatic plants, possess potential anti-cancer properties (5). Their major bioactive constituents include monoterpenes, sesquiterpenes, phenolics and oxygenated derivatives such as thymol, carvacrol, limonene, eugenol, linalool, β-caryophyllene and α-pinene (6). These compounds exhibit anti-proliferative, pro-apoptotic, anti-inflammatory, antioxidant, anti-metastatic and chemosensitising activities (7).

EOs fight cancer in several ways. Some of their molecules can stop the cell cycle, thereby blocking cell growth and triggering cell death (8). Moreover, EOs affect cell signalling and inhibit angiogenesis, both of which are essential for tumour enrichment (9). As a result, EOs can cause oxidative damage in tumour cells by upsetting their redox balance. Some compounds can change the physical and chemical properties of the cell membrane. This can make the membrane unstable and cause the cell to lose its normal function (10). Developing evidence suggests that EOs and their bioactive constituents can effectively target CSCs by modulating multiple signalling pathways involved in stemness and survival (11).

EOs block the Wnt/β-catenin pathway, which is important for CSC self-renewal. They do this by lowering β-catenin activity and decreasing the levels of stem cell markers like CD44 and ALDH1 (12). Phenolic compounds have been shown to reduce spheroid formation and impair CSC viability (13). Furthermore, clove EO constituents suppress Notch signalling by downregulating Notch1 and Hes1 expression, thereby reducing CSC proliferation, enhancing differentiation and increasing sensitivity to chemotherapy (14). EO-derived terpenoids also target the Hedgehog signalling pathway by inhibiting GLI1 and Smoothened (SMO), thereby restricting CSC renewal and tumour growth (15). Moreover, EOs inhibit epithelial–mesenchymal transition (EMT) by downregulating mesenchymal markers and restoring E-cadherin expression (**Table 1**). Compounds such as terpenes further inhibit migration and invasion by suppressing matrix metalloproteinases (31). Another important mechanism of EOs action involves disruption of CSC redox balance by raising the levels of reactive oxygen species (ROS) inside cells, which causes DNA damage, disrupts mitochondria, activates caspases, and leads to cell death (32). Recent evidence suggests that EOs can modulate CSC signalling pathways and overcome multidrug resistance (MDR), thereby improving therapeutic efficacy (33).

**Table 1:** Essential oils (EOs) currently under extensive investigation for their anti-cancer properties.

Essential oil	Bioactive constituent(s)	Cancer type(s)	Mechanism of action	Drug resistance target	Current status	Ref
Clove (*Syzygium aromaticum*)	Eugenol	Breast, colon, lung, melanoma, oral cancers	Induces apoptosis, cell-cycle arrest, ROS generation, inhibits angiogenesis	Suppresses Notch signalling, decreases Hes1, reverses ABC transporter-mediated resistance, sensitises to chemotherapy	Preclinical	(14, 16)
Thyme (*Thymus vulgaris*)	Thymol	Breast, colorectal, prostate	Mitochondrial apoptosis, ROS generation, anti-inflammatory	Reduces Nanog, Sox2, Oct4 expression; inhibits ABC transporters; enhances chemosensitivity	Preclinical	(17, 18)
Oregano (*Origanum vulgare*)	Carvacrol	Breast, colon, liver, gastric	Apoptosis induction, oxidative stress, inhibition of proliferation	Inhibits ABC drug-efflux pumps, suppresses CSC markers, increases intracellular drug accumulation	Preclinical	(19, 20)
Black Pepper (*Piper nigrum*)	β-Caryophyllene	Breast, colorectal, pancreatic	Anti-inflammatory, apoptosis, immune modulation	Modulates cannabinoid receptor signalling, reduces CSC stemness, inhibits Wnt/PI3K signalling	Preclinical	(21, 22)
Citrus oils (*Citrus limon, C. sinensis*)	Limonene	Breast, liver, pancreatic	Cell-cycle arrest, apoptosis, antioxidant modulation	Suppresses CSC proliferation and EMT	Preclinical	(23)
Lavender (*Lavandula angustifolia*)	Linalool	Lung, breast, leukaemia	Mitochondrial dysfunction, ROS-mediated apoptosis	Reduces CSC survival and enhances chemotherapy response	Preclinical	(24)
Tea Tree (*Melaleuca alternifolia*)	Terpinen-4-ol	Melanoma, breast	Membrane disruption, apoptosis, oxidative stress	Potential inhibition of CSC viability and proliferation	Preclinical	(25, 26)
Lemongrass (*Cymbopogon citratus*)	Citral	Breast, prostate, colon	Induces apoptosis, inhibits proliferation, cell-cycle arrest	Reduces EMT and stem-cell characteristics	Preclinical	(27)
Pine (*Pinus spp*.)	α-Pinene, β-Pinene	Lung, liver, gastric	Anti-proliferative, ROS generation, apoptosis	Possible inhibition of migration and CSC maintenance	Preclinical	(28)
Geranium (*Pelargonium graveolens*)/Rose oil	Geraniol	Colon, pancreatic, liver	Cell-cycle arrest, apoptosis, anti-metastatic activity	May suppress PI3K/Akt signalling and CSC-associated pathways	Preclinical	(29, 30)

Whilst substantial preclinical evidence suggests that EOs and their bioactive constituents can target CSC-associated signalling pathways and enhance the sensitivity of resistant tumour cells to conventional therapies, clinical translation remains limited. The majority of existing data originates from *in vitro* studies and animal models, with only minimal clinical evidence supporting efficacy and safety in cancer patients. This significant lack of clinical phase studies constitutes a major limitation in the field and must be considered when evaluating the therapeutic potential of EOs. Consequently, the findings presented in this review should be regarded as promising preclinical evidence rather than established clinical practice. Addressing this translational gap will require standardised EO formulations, comprehensive pharmacokinetic and toxicological assessments and rigorously designed, adequately powered clinical trials to determine safety, optimal dosing and efficacy as adjuncts to conventional anti-cancer therapies.

## 2 Association of CSCs and drug resistance

**Figure 1:**
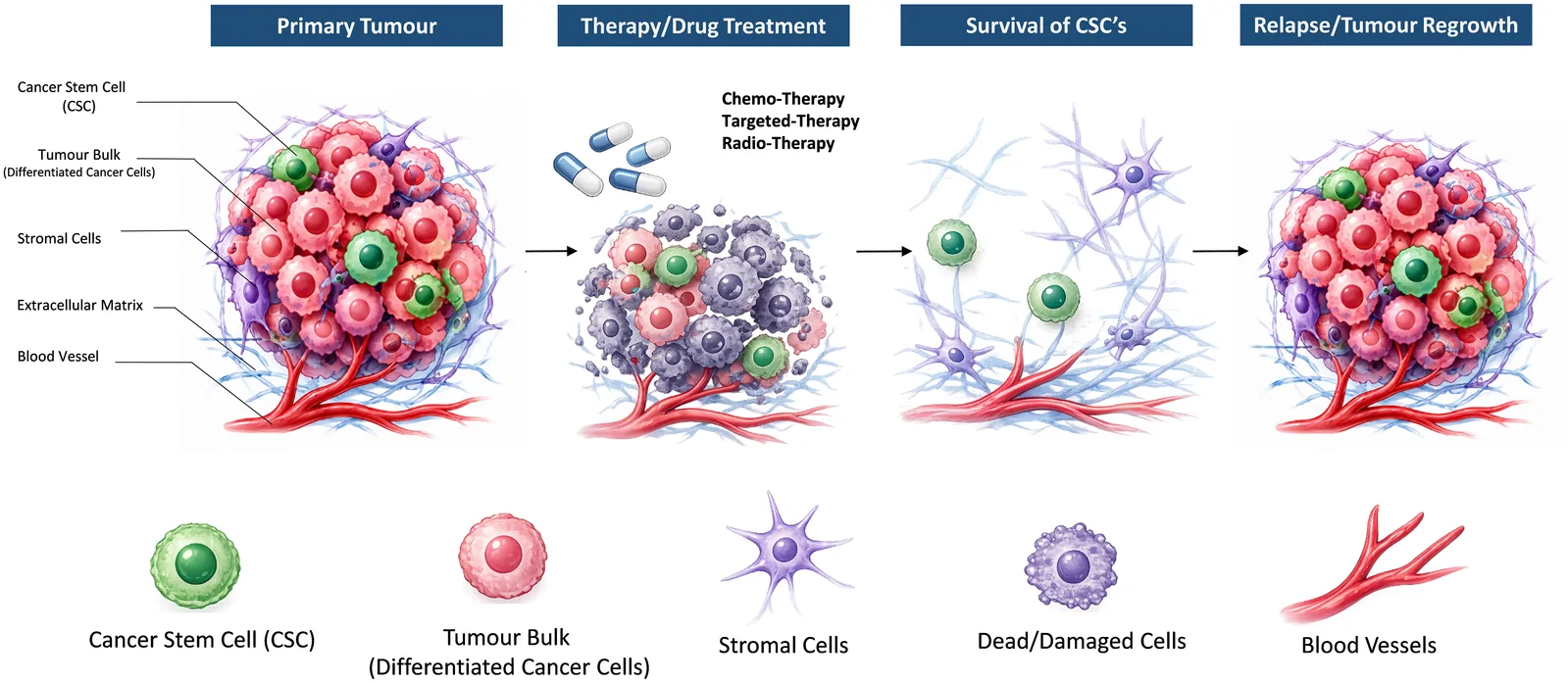
Overview of CSC-driven tumour relapse and drug resistance. This illustrates the fate of cancer stem cells (CSCs) during cancer therapy. Whilst chemotherapy, targeted therapy or radiotherapy effectively eliminates most differentiated tumour cells, a small population of CSCs survives. These surviving CSCs retain self-renewal capacity, driving tumour regrowth, metastasis, therapeutic resistance and disease relapse, thereby contributing to poor clinical outcomes.

CSCs represent a specialised and highly tumorigenic subset of cancer cells (34). These cells exhibit properties analogous to normal stem cells, such as self-renewal, multilineage differentiation potential and sustained proliferative capacity (35). As a result, these cells are widely recognised as major contributors to tumour initiation, progression, metastasis and disease recurrence (**Figure 1**). By contrast, differentiated cancer cells constitute the majority of a tumour mass, whereas CSCs occupy the apex of a hierarchical cellular organisation and generate heterogeneous progeny that drive intratumoral diversity (36).

The emergence of the CSC concept has greatly changed the traditional view of cancer development. Whereas earlier models regarded tumours as relatively homogeneous populations of malignant cells with comparable tumour-forming potential, the CSC paradigm proposes that only a limited fraction of cells within a tumour possesses the capacity for long-term maintenance and regeneration of the malignancy (37). This ranked organisation provides a plausible explanation for the frequent observation of tumour relapse following apparently successful treatment. Accordingly, although conventional therapies may effectively eliminate most proliferating cancer cells, CSCs often survive and retain the ability to re-establish tumour growth, resulting in recurrence and disease progression (38).

One of the primary obstacles in cancer therapy is the inherent resistance of CSCs to various anti-cancer agents. Many chemotherapeutic drugs preferentially target rapidly dividing cells; however, CSCs frequently reside in a dormant or slow-cycling state, rendering them less susceptible to such treatments (39). Furthermore, CSCs possess highly efficient cellular defence mechanisms that promote survival under therapeutic stress. These comprise enhanced DNA repair capacity, increased antioxidant activity to counteract reactive oxygen species, metabolic flexibility supported by mitochondrial adaptations, and elevated expression of anti-apoptotic proteins that prevent programmed cell death (40).

A widely studied mechanism underlying CSC-mediated drug resistance is the upregulation of ATP-binding cassette (ABC) transporter proteins. Members of this family, such as ABCB1 (P-glycoprotein), ABCG2, and ABCC1 (MRP1), actively export a broad spectrum of chemotherapeutic agents from the intracellular environment (41). As a result, drug accumulation within CSCs is markedly reduced, preventing the attainment of cytotoxic concentrations and aiding the development of multidrug resistance.

The tumour microenvironment additionally enhances CSC survival and therapeutic resistance (42). Factors such as hypoxia, acidic conditions, chronic inflammation and cancer-associated fibroblasts, infiltrating immune cells and extracellular matrix components jointly establish a sustaining niche that preserves CSC properties (43). Signals originating from this microenvironment activate multiple molecular pathways that sustain stemness, promote epithelial–mesenchymal transition (EMT), facilitate immune escape and increase treatment resistance. Consequently, CSCs remain a critical therapeutic target and strategies intended to eliminate this cell population are considered necessary for preventing relapse, overcoming drug resistance and achieving durable cancer remission (44).

## 3 Cancer stem cell signalling and target genes

**Figure 2:**
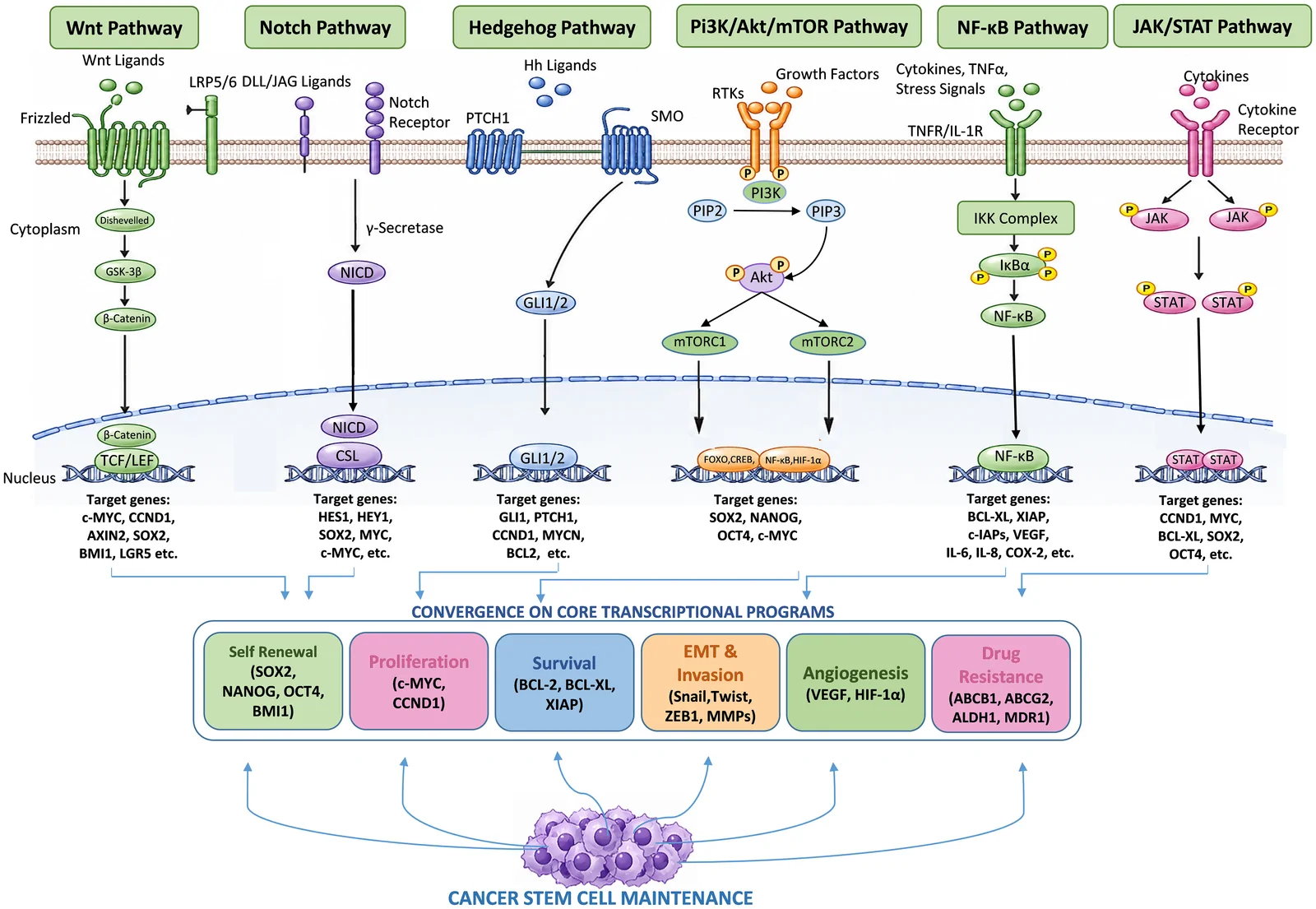
Major signalling pathways regulating cancer stem cell (CSC) maintenance and therapeutic resistance. The schematic illustrates the principal signalling pathways involved in CSC biology, including Wnt/β-catenin, Notch, Hedgehog, PI3K/Akt/mTOR, NF-κB, and JAK/STAT. Activation of these pathways regulates the transcription of genes controlling self-renewal, proliferation, survival, epithelial–mesenchymal transition (EMT), angiogenesis, and multidrug resistance.

CSCs are maintained by a complex, tightly regulated network of signalling pathways that preserve their stem-like properties and enable rapid adaptation to therapeutic interventions and changes in the tumour microenvironment. Both endogenous and exogenous genes, as well as microRNAs, modulate these pathways. These signalling cascades induce downstream gene expression in CSCs, including cytokines, growth factors, apoptosis related genes, anti-apoptotic genes, proliferation genes, and metastasis associated genes. Furthermore, signalling networks coordinate essential biological processes such as self-renewal, aberrant differentiation, phenotypic plasticity, metabolic reprogramming, tumour initiation, invasion, metastasis and resistance to chemotherapy and radiotherapy. The Wnt/β-catenin, Notch, Hedgehog, JAK/STAT, NF-κB, and PI3K/AKT/mTOR pathways are amongst the most prominent, acting as central regulators of CSC maintenance, lineage commitment, survival, and proliferation (**Figure 2**). These pathways do not function in isolation, instead, they engage in extensive crosstalk, forming an integrated signalling network in which the activation or inhibition of one pathway can influence the activity of others. These interactions converge on shared transcription factors, epigenetic regulators and metabolic programs that collectively determine CSC fate and support long-term survival (45). Furthermore, these signalling cascades continuously integrate extracellular cues, including nutrient availability, hypoxia, inflammatory mediators and oxidative stress. This integration enables CSCs to maintain cellular plasticity and adapt to adverse conditions. The resulting adaptive signalling landscape contributes to the intrinsic resistance of CSCs to conventional anti-cancer therapies, as well as to disease recurrence and metastatic dissemination. Understanding the coordinated roles and interactions amongst the Wnt/β-catenin, Notch, Hedgehog, PI3K/AKT/mTOR, NF-κB and JAK/STAT pathways provides critical insight into the molecular mechanisms that govern CSC biology. This knowledge supports the development of targeted therapeutic strategies designed to disrupt these interconnected signalling hubs, thereby overcoming treatment resistance and improving clinical outcomes.

### 3.1 Wnt/β-catenin signalling enhances the self-renewal capacity of CSCs

Wnts are large protein ligands that regulate diverse cellular processes, including cell polarity and cell fate determination (46). Aberrant regulation of both canonical and non-canonical WNT signalling is observed in numerous human malignancies (47). Consequently, mutations within the Wnt pathway contribute significantly to the initiation of various cancers (48). Dysregulated Wnt/β-catenin signalling also mediates cancer cell resistance to cytotoxic chemotherapy and serves as a key regulator of CSC biology (49, 50). Leucine-rich repeat containing G protein-coupled receptor 5 (LGR5) is a cell surface GPCR that binds R-spondin ligands in conjunction with Wnt ligands, thereby activates the Frizzled/LRP6 complex. Overactivation of Wnt signalling leads to nuclear accumulation of β-catenin, which subsequently activates target genes such as c-MYC, AXIN2, SOX2, BML1, CCND1 and LGR5 that promote cell growth, survival and stemness (51) (**Figure 2**). Therefore, LGR5 and R-spondin ligands are critical modulators of Wnt signalling (52). LGR5 was recently identified as a CSC marker (53) and is upregulated in several human cancers, including breast, gastric, cervical and colorectal cancers (54–57). Notably, LGR5 promotes CSC self-renewal and tumorigenesis in breast cancer through activation of Wnt/β-catenin signalling (54). Additionally, increased LGR5 expression in cervical CSCs enhances tumour sphere-forming efficiency and confers resistance to cisplatin treatment (56).

### 3.2 Notch signalling pathway in cancer stem cell development

The Notch pathway is integral to CSC maintenance, therapy resistance and TME interactions, particularly in GBM, breast, pancreatic and colorectal cancers (58). Activation of Notch signalling in CSCs correlates with increased tumorigenicity and resistance to chemotherapy and radiation (59). The pathway is initiated when ligands such as Jagged or Delta-like proteins on one cell bind to Notch receptors on an adjacent cell, triggering the release of the Notch intracellular domain. This domain translocates to the nucleus and regulates gene expression (60) (**Figure 2**). In oncogenic contexts, dysregulated Notch signalling expands the CSC population, maintains their undifferentiated state and enhances treatment resistance by upregulating HES1, SOX2, cMYC, and HEY1 (61). Multiple studies have demonstrated that Notch pathway activation in CSCs promotes cell survival, self-renewal and metastasis whilst inhibiting apoptosis. Aberrant Notch signalling further supports self-renewal and metastasis in breast and hepatocellular carcinoma (HCC) stem cells (62, 63). In CSCs, Notch overactivation is associated with sustained proliferation, inhibition of differentiation and increased expression of prosurvival genes (64).

### 3.3 Hedgehog signalling pathway in cancer stem cell progression

The hedgehog (Hh) signalling pathway plays a pivotal role in maintaining the stemness of CSCs. This signalling is another developmental pathway that tumours often use to their advantage. Normally, Hedgehog signalling helps shape embryos and repair tissues. In cancer, abnormal activation of this pathway helps CSCs survive, supports tumour growth and promotes spread.

The Hh signalling pathway comprises ligands and receptors, forming a complex network that includes extracellular Hh ligands, the transmembrane receptor Patched (PTCH), the transmembrane protein Smoothened (SMO), intermediate transduction molecules and the downstream effector GLI (65). The canonical Hh pathway involves several cascades: PTCH binds to the Hh ligand, which relieves its inhibition of SMO. This process enables the dissociation of the Suppressor of Fused (SuFu) from GLI, allowing GLI activators to regulate target gene expression (66) (**Figure 2**). Each component of the Hh pathway has a distinct regulatory function; SMO acts as a positive regulator, whereas PTCH serves as a negative regulator. Upon ligand binding, Hh ligands interact with PTCH receptors on the cell membrane, initiating intracellular signal transduction (67). The expression of Hh signalling components is elevated in CSCs. For instance, Hh signalling supports the maintenance, proliferation, self-renewal and tumorigenicity of lung adenocarcinoma stem cells (68). Additionally, Hedgehog proteins contribute to metabolic regulation by promoting stem-like characteristics and enabling CSCs to adapt to various environmental pressures, including energetic and mechanical challenges.

### 3.4 PI3K/Akt/mTOR signalling pathway promotes stemness and resistance

The PI3K/AKT/mTOR pathway is a central regulator of cell growth, survival and metabolism. Activation is initiated when growth factors or cytokines bind to receptor tyrosine kinases, stimulating PI3K to convert PIP2 to PIP3 at the plasma membrane. PIP3 subsequently recruits and activates AKT, which phosphorylates downstream targets that drive cell proliferation, angiogenesis and metabolic reprogramming (69). A key downstream effector of AKT is mTOR, a kinase that forms two distinct complexes: mTORC1 and mTORC2. mTORC1 primarily governs protein synthesis and autophagy, whilst mTORC2 modulates cytoskeletal organisation and AKT regulation (70). In CSCs, hyperactivation of PI3K/AKT/mTOR signalling is frequently associated with increased expression of cell-cycle regulators and core stemness transcription factors such as OCT4, SOX2 and NANOG (71, 72) (**Figure 2**). The activation of mTORC1 versus mTORC2 is context dependent and regulated by upstream signalling dynamics and subcellular localisation. mTORC1 activation typically requires amino acid availability and RHEB mediated recruitment to the lysosomal membrane, whereas mTORC2 assembly is stimulated by growth factor signalling through PI3K and subsequently activates AKT via phosphorylation at Ser473 (73). In CSCs, dysregulation of this pathway is often linked to genetic alterations, including activating mutations in PIK3CA or loss-of-function mutations in PTEN, both of which enhance PI3K/AKT/mTOR signalling (74, 75). These mutations upregulate stemness-associated genes and confer resistance to apoptosis, thereby supporting CSC maintenance and evasion of therapy. Collectively, these molecular events contribute to chemotherapy resistance, enhance tumour initiation capacity, and enable CSCs to adapt metabolically to adverse microenvironments.

### 3.5 NF-κB signalling pathway maintains and promotes CSC stemness

Inflammation related pathways, including NF-κB, play crucial roles in CSCs. The NF-κB pathway comprises multiple signalling cascades and is activated by diverse cellular stimuli, resulting in IκB kinase activation and subsequent degradation of the I-kappa B (IκB) protein. This process releases NF-κB dimers, which undergo post-translational modifications and translocate to the nucleus. In the nucleus, these dimers bind to specific target genes and promote the transcription of genes such as BCL-XL, XIAP, c-IAF, COX-2, IL6, and IL8 (76) (**Figure 2**). NF-κB activation is essential for the formation of breast CSCs (77). Pathway analysis of CSCs isolated from patients with prostate cancer and non-small cell lung cancer (NSCLC) has revealed specific activation of the NF-κB pathway, which is also implicated in the maintenance of stemness (78). Activated NF-κB in CSCs is closely associated with tumorigenesis and metastasis. In ovarian CSCs, NF-κB pathway-related proteins are highly expressed, and inhibition of this pathway reduces the CSC population (79). In CD133+ liver CSCs, upregulated BMI-1 enhances NF-κB activation and nuclear translocation, thereby promoting CSC stemness and metastatic potential whilst inhibiting apoptosis (80).

### 3.6 JAK/STAT signalling pathway maintains CSC viability

The JAK/STAT signalling pathway comprises tyrosine kinase-associated receptors that receive extracellular signals, the tyrosine kinase JAK that transmits these signals, and the transcription factors STAT (81). Upon ligand binding to the receptor, JAK is phosphorylated and activated, thereby recruiting and phosphorylating STAT. The phosphorylated STAT forms dimers and translocates to the nucleus, where it binds to target genes and BCL-XL, thereby regulating the expression of downstream genes such as SOX-2, OCT-4, and MYC (82) (**Figure 2**). Regulation of the JAK/STAT pathway is closely associated with the maintenance of stemness. Selective inhibition of STAT3 has been shown to significantly reduce the expression of stemness-related genes in breast CSCs (83). Activation of the STAT pathway supports the maintenance of stemness in osteosarcoma, liposarcoma and thyroid cancer (84–86). Immune mechanisms may play a significant role in modulating the JAK/STAT pathway in CSCs. Furthermore, regulation of the JAK/STAT pathway in CSCs is intricately associated with tumorigenesis, metastasis, and metabolic reprogramming. In prostate CSCs, IL-6-mediated activation of the JAK/STAT pathway is a critical event in tumorigenesis and its inhibition abolishes tumour initiation (87).

In summary, CSC signalling pathways form a complex, overlapping network. Because these pathways interact, targeting only one pathway often fails, as it can increase resistance and reduce flexibility. Treatments that target multiple at the same time may be more effective at eliminating CSCs.

## 4 Mechanisms of stem cell-mediated drug resistance

**Figure 3:**
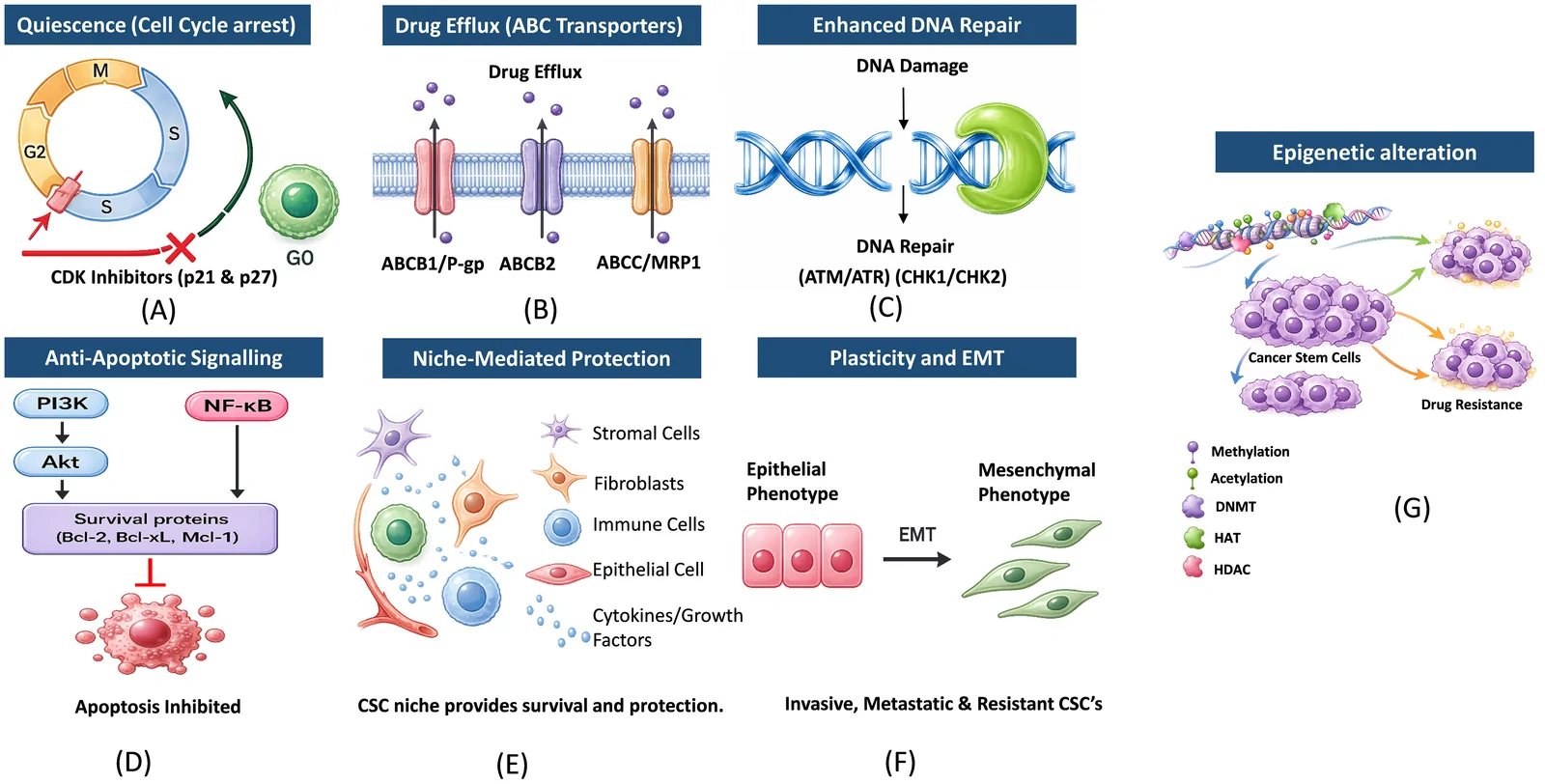
Key molecular mechanisms underlying cancer stem cell (CSC)-mediated drug resistance. The schematic summarises the major mechanisms by which CSCs evade anti-cancer therapies. These include **(A)** cellular quiescence, allowing CSCs to escape treatments targeting proliferating cells; **(B)** overexpression of ATP-binding cassette (ABC) transporters that promote drug efflux; **(C)** enhanced DNA damage repair pathways that preserve genomic integrity; **(D)** activation of anti-apoptotic signalling pathways that inhibit programmed cell death; **(E)** niche-mediated protection through interactions with stromal, immune, endothelial, and extracellular matrix components; **(F)** epithelial–mesenchymal transition (EMT) and cellular plasticity, which enhance invasion, metastasis, and stemness; and **(G)** epigenetic alterations, including DNA methylation, histone modifications, and non-coding RNAs, which regulate gene expression to sustain CSC maintenance and therapeutic resistance. Collectively, these mechanisms contribute to tumour persistence, disease recurrence, and multidrug resistance.

CSCs demonstrate intrinsic resistance to radiotherapy and chemotherapy, rendering them less responsive to conventional anti-cancer treatments than the bulk tumour cell population (88). Most cytotoxic agents and radiotherapy primarily target rapidly proliferating, apoptosis-sensitive differentiated cancer cells, whereas the relatively quiescent CSC population remains largely unaffected. As a result, treatment often selectively enriches therapy-resistant CSCs, which contributes to tumour relapse and disease progression (89, 90). CSCs display notable plasticity, heterogeneity and adaptability to changing microenvironmental conditions. Multiple intrinsic mechanisms underlie their therapy resistance, including slow cell-cycle kinetics, activation of anti-apoptotic proteins such as Bcl-2 and Mcl-1, efficient DNA damage repair mediated by DNA checkpoint kinases, and sustained expression of stemness-associated pathways (91). After radiotherapy, CSCs can repair DNA damage through transient cell-cycle arrest, enabling them to either undergo apoptosis or re-enter the cell cycle and repopulate the tumour (92). Beyond intrinsic mechanisms, CSC resistance is also shaped by extrinsic factors, particularly the tumour microenvironment (TME). The TME constitutes a complex multicellular niche in which dynamic interactions amongst stromal, immune, endothelial and cancer cells regulate tumour progression and therapeutic response (93, 94). These interactions foster genetic and epigenetic diversity within tumours and support CSC maintenance and plasticity. Mesenchymal-like invasive CSCs are often located at tumour margins in regions of high cellular density, where they interact closely with TME components (95, 96) (**Figure 3**). This specialised niche is essential for preserving CSC stemness, survival and invasive potential, whilst disruption of these interactions significantly reduces their functional capacity. The capacity of CSCs to evade treatment and repopulate tumours highlights the necessity for therapeutic strategies that target multiple survival pathways, rather than focusing exclusively on rapidly proliferating cancer cells. These resistance traits present a major barrier to treatment success, contributing to tumour recurrence and metastasis even after initially effective interventions.

### 4.1 Alteration in cell cycle

CSCs contribute significantly to drug resistance through alterations in cell cycle regulation, allowing them to survive chemotherapy and regenerate tumours after treatment (2) (**Figure 3A**). Despite the bulk of rapidly proliferating cancer cells, CSCs often remain in a quiescent (G0/G1) state, rendering them less vulnerable to cell cycle-dependent chemotherapeutic agents such as 5-Fluorouracil and Cisplatin (97). In addition, dysregulated expression of cyclins, cyclin-dependent kinases (CDKs) and CDK inhibitors (p21 and p27), along with activation of stemness-associated signalling pathways including Wnt/β-catenin, PI3K/Akt, Notch, and Hedgehog, supports CSC survival, self-renewal, and regulated re-entry into the cell cycle after treatment (98). These adaptive strategies allow CSCs to evade cytotoxic therapies, promote tumour recurrence and metastasis, and constitute promising therapeutic targets for addressing cancer drug resistance.

### 4.2 Drug efflux transporters

A common way cancer cells resist drugs is by overexpressing ATP-binding cassette (ABC) transporters, including ABCB1, ABCC1 and ABCG2 (99). These proteins use ATP-derived energy to pump many anti-cancer drugs out of the cell, preventing them from reaching effective levels (**Figure 3B**). This process is especially important in CSCs and is often associated with the side population phenotype observed in dye-exclusion tests (37).

### 4.3 Enhanced DNA repair

Many cancer treatments work by damaging the DNA of cancer cells. Cancer cells may develop resistance by enhancing their DNA repair capacity through pathways including nucleotide excision repair, base excision repair, homologous recombination, and non-homologous end joining (100). In response to therapeutic stress, CSCs activate DNA damage checkpoint pathways, including ATM/ATR and CHK1/CHK2, which facilitate efficient DNA repair and prevent apoptosis (101) (**Figure 3C**). With better DNA repair, cancer cells can fix damage that would normally kill them and keep growing. This is a key reason for resistance to drugs like platinum compounds, alkylating agents, topoisomerase inhibitors and radiation (41).

### 4.4 Apoptosis avoidance

Resistant cancer cells exhibit a reduced propensity for programmed cell death, known as apoptosis. This resistance often results from increased production of anti-apoptotic proteins, including Bcl-2, Bcl-xL, Mcl-1 and surviving (**Figure 3D**), or decreased expression of pro-apoptotic proteins such as Bax, Bak and caspases (102). Additionally, mutations in the TP53 gene impair the cellular response to DNA damage, enabling cancer cells to persist despite intensive therapeutic interventions (37).

### 4.5 Tumour microenvironment

Drug resistance is affected not just by the cancer cells themselves, but also by the tumour microenvironment, which sends signals that help protect cancer cells from treatment. Cells such as cancer-associated fibroblasts, endothelial cells, and immunosuppressive cells, along with proteins in the surrounding matrix and factors like IL-6, TGF-β and TNF-α, all contribute to this protection (103) (**Figure 3E**). Low oxygen levels in tumours activate HIF-1α, which alters how cells use energy, increases signals for new blood vessel formation, and helps cancer cells become more stem-like and resistant (35, 41, 104).

### 4.6 Epithelial-mesenchymal transition

Epithelial-mesenchymal transition (EMT) is a biological process during which epithelial cells lose their cell-cell junctions and cell-matrix attachments, subsequently acquiring mesenchymal characteristics, including enhanced motility, invasiveness and resistance to stress (105). EMT is linked to cancer spreading, avoiding the immune system, and gaining stem cell-like traits (106) (**Figure 3F**). Cells that go through EMT often become more resistant to chemotherapy and targeted treatments because they are better able to survive and adapt (35).

### 4.7 Epigenetic alterations

Epigenetic modifications, including DNA methylation, histone acetylation or deacetylation may silence tumour suppressor genes or activate resistance-associated pathways without altering the underlying DNA sequence (107) (**Figure 3G**). Because these changes can be reversed, they are promising targets for therapy. Epigenetic flexibility also helps cancer cells quickly adapt to drug-induced stress (37). Since resistance has many causes, effective treatments usually require combining multiple approaches that target both the cancer cells themselves and the surrounding microenvironment (37, 41).

## 5 Chemical composition and anti-cancer potential of EOs

EOs are concentrated, volatile mixtures made from aromatic plants. They are usually extracted by steam distillation, hydrodistillation, cold pressing, or newer methods like supercritical fluid extraction (108). The chemical makeup of EOs, which affects their biological activity, can vary with plant species, chemotype, where and how the plant was grown, when it was harvested, how it was stored, and the extraction method used. Most of the chemicals in EOs are terpenes and terpenoids, but they also contain smaller amounts of phenylpropanoids, alcohols, aldehydes, ketones, esters and oxides (109). Some of the main components associated with anti-cancer effects particularly CSC’s are limonene, α-pinene, β-pinene, linalool, geraniol, thymol, carvacrol, eugenol, citral, β-caryophyllene and terpinen-4-ol (110) (**Table 1**). These substances can work on their own or together within the oil.

**Figure 4:**
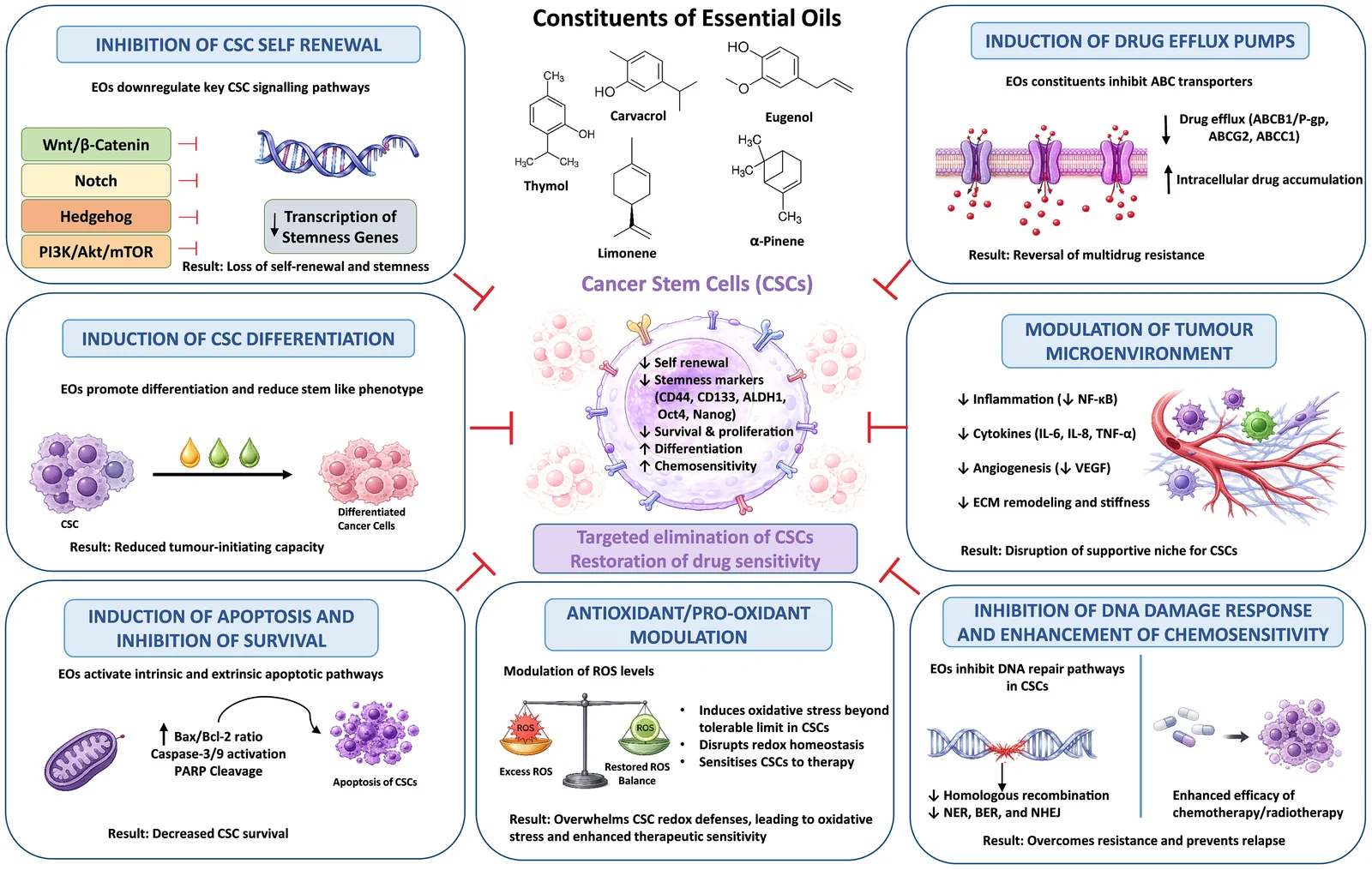
Mechanisms by which essential oils target cancer stem cells (CSCs) and overcome drug resistance. Representative Essential oils (EOs) constituents like thymol, carvacrol, eugenol, limonene, and α-pinene constituents inhibit CSC self-renewal by suppressing Wnt/β-catenin, Notch, Hedgehog, and PI3K/Akt/mTOR signalling, promote CSC differentiation, induce apoptosis, inhibit ABC transporter-mediated drug efflux, disrupt the tumour microenvironment, impair DNA damage repair and modulate reactive oxygen species (ROS) homeostasis.

EOs have gained attention in cancer research for their numerous antitumour effects in laboratory studies (111). Numerous EO components induce cell death by disrupting the mitochondrial membrane, facilitating cytochrome c release, and activating caspases (112). Others inhibit cancer cells from dividing by causing cell cycle arrest (29). In some cases, EOs increase the amount of reactive oxygen species (ROS) inside cells, which can cause damage and cell death through oxidative stress (113). EOs can inhibit angiogenesis (114), decrease the activity of specific enzymes, modulate epithelial-mesenchymal transition (EMT) (115), reduce inflammatory mediators such as NF-κB and COX-2 (116), and enhance the immune response against tumours (5) (**Figure 4**). Some EO components have shown they can target cancer cells whilst leaving normal cells unharmed, but this effect is not always consistent and should be viewed with caution (111).

Despite the growing body of evidence supporting the anti-cancer activity of EOs and their ability to target CSCs, their clinical translation remains in its infancy. Although numerous *in vitro* and preclinical studies have shown that EOs can suppress CSC self-renewal, inhibit EMT, modulate stemness-associated signalling pathways, and enhance the efficacy of conventional chemotherapeutic agents. Simultaneously, several scientific, technical and regulatory challenges continue to impede their development as clinically approved anti-cancer therapeutics. These challenges primarily arise from compositional variability, limited standardization, uncertain safety and pharmacokinetic profiles, delivery challenges, insufficient clinical evidence and regulatory complexities associated with EOs.

## 6 EOs as modulators of cancer stemness

EOs may be especially useful for targeting CSCs, which often cause recurrence, metastasis, and treatment failure. More studies in laboratory and animal models show that compounds in EOs can disrupt the signalling pathways that support CSC survival and function (5, 35, 111, 117). Some terpenoids and phenolic compounds from EOs can block signalling pathways like Wnt/β-catenin, Hedgehog, Notch, and PI3K/Akt. Blocking these signalling pathways may reduce CSC’s ability to self-renew, promote their maturation and increase their sensitivity to treatment (**Table 1**). For instance, B-caryophyllene modulates cannabinoid receptor signalling and reduces stemness-associated traits (118). Thymol and carvacrol have also been shown in experiments to reduce the activity of transcription factors, including NANOG, SOX2, and OCT4, which control pluripotency and stem-like behaviour (5, 35, 117, 119). Researchers often test CSC activity by measuring how well they form spheres, such as tumour spheres, mammospheres, or colonospheres, which show their ability to renew without attaching to a surface. EOs and their compounds have been shown to reduce both the number and size of these spheres across various cancer models, suggesting they can limit CSC function (120). After treatment with certain essential oil components, levels of common CSC surface markers, such as EpCAM, ALDH1, CD133, and CD44, also decrease (5, 35, 117, 119). EOs can also affect processes like EMT and CSC plasticity. By reversing EMT or blocking the pathways that cause it, EOs may reduce the number of new CSCs formed from other cancer cells. They can also change oxidative stress and inflammation inside cells, making it harder for CSCs to survive (5, 35, 117, 121). Whilst these results are promising, most of the evidence comes from early-stage research. The effects of EOs on CSCs need to be confirmed through more rigorous animal studies, standardised experiments, and eventually, clinical trials. Before these oils can be used as treatments, it is important to optimise doses, develop effective delivery methods, assess toxicity, and ensure consistent oil composition (5, 111).

## 7 Influence of EOs on drug resistance

EOs are increasingly being investigated as chemosensitisers, meaning agents that improve the responsiveness of resistant cancer cells to standard therapy. This is one of their most promising applications, particularly in multidrug-resistant tumours, where standard treatments lose efficacy over time (5, 111).

### 7.1 Inhibition of drug efflux pumps

Some components of EOs can block ABC transporters, which helps keep chemotherapy drugs inside cancer cells (122). Compounds such as carvacrol, thymol, and eugenol have been shown to reduce drug export in experimental studies (123) (**Table 1**) (**Figure 4**). By limiting this export, these molecules may help cancer cells become sensitive again to treatments that had stopped working (5, 37, 41).

### 7.2 Induction of apoptosis

Resistant cancer cells often avoid cell death, even when exposed to strong treatments. EOs such as Eugenol, Thymol, Carvacrol, Limonene, Linalool, Citral, and Geraniol may help address this by modulating Bcl-2 proteins, increasing mitochondrial permeability, activating caspases, and restoring cell death signalling (**Table 1**) (**Figure 4**). This ability to trigger apoptosis is a key reason why EOs can work well with chemotherapy (5, 111).

### 7.3 Modulation of cellular signalling

EOs, especially Geraniol, β-Caryophyllene and Eugenol, have been shown to disrupt key survival pathways in cancer cells, such as PI3K/Akt, NF-кB, STAT3, and MAPK (5) (**Table 1**). These pathways are often responsible for self-renewal and stemness, so blocking them may reduce survival signals, diminish stem cell-like properties, limit inflammation, and weaken stress responses (5, 35, 117, 119) (**Figure 4**).

### 7.4 Regulation of oxidative stress

Many resistant cancer cells have strong antioxidant defences, which help them survive the reactive oxygen species (ROS) caused by treatment (**Figure 4**). Some EOs like Eugenol, Thymol, Carvacol, Linalool, Terpinen-4-ol, α-Pinene and β-Pinene components can increase ROS to harmful levels or weaken these defences, pushing the cells towards death. This effect can be even stronger when combined with treatments that already cause oxidative stress (5, 111).

### 7.5 Epigenetic effects

More research suggests that EO components may affect epigenetic regulation, including histone modifications, DNA methylation, and the activity of certain non-coding RNAs associated with resistance (124–126). Whilst this field is still new, it could help explain how natural compounds might change resistant cancer cells (37, 41, 121).

### 7.6 Combination therapу

Combining EOs with standard cancer drugs may be their most useful application (5, 127). Studies have found that EO components can interact with drugs such as doxorubicin, cisplatin, paclitaxel and 5-fluorouracil (32, 127–129). This teamwork might allow for lower doses, less toxicity, and better cancer control. In theory, using EOs alongside regular treatments could help overcome resistance whilst keeping the benefits of current therapies (5, 111). However, there are still some important limitations. EOs have complex chemistry and might interact with standard drugs in unexpected ways. We do not yet fully understand how they are processed in the body or how safe they are for regular use in cancer treatment. For now, EOs should be considered experimental additions, not proven cancer therapies (5, 111).

## 8 Challenges and future perspectives for the clinical translation of EOs in cancer therapy

Although EOs demonstrate considerable potential as anti-cancer agents, several critical challenges must be resolved before clinical translation can occur. A primary obstacle is the inherent chemical heterogeneity of EOs. As complex mixtures of volatile compounds, their chemical composition varies substantially according to plant species, geographical origin, climatic conditions, harvesting season, plant maturity, extraction method and storage conditions. Consequently, oils derived from the same botanical species may exhibit significant differences in the concentrations of biologically active constituents, including monoterpenes, sesquiterpenes and phenylpropanoids (130, 131). This variability compromises batch-to-batch consistency, reproducibility of experimental findings and the accurate interpretation of pharmacological outcomes. Inconsistencies in analytical methodologies and the absence of universally accepted quality control standards further hinder cross-study comparisons. Therefore, rigorous chemical characterisation using standardised analytical techniques, such as gas chromatography–mass spectrometry (GC–MS), in conjunction with the implementation of Good Agricultural and Collection Practices (GACP) and Good Manufacturing Practices (GMP), is essential to ensure reproducible biological activity and to facilitate regulatory approval (132, 133).

A further major challenge is the unfavourable pharmacokinetic profiles of many EO constituents. Most volatile phytochemicals possess poor aqueous solubility, limited oral bioavailability, rapid gastrointestinal absorption with extensive first-pass metabolism, short systemic half-lives, chemical instability and rapid elimination. These characteristics often hinder the maintenance of therapeutically effective concentrations at tumour sites, thereby restricting clinical efficacy despite encouraging *in vitro* findings. Moreover, many EO components are highly susceptible to oxidation, volatilisation, photodegradation, and thermal degradation during storage and formulation, which further diminishes their stability and therapeutic potential. Overcoming these limitations requires the development of advanced drug-delivery systems, including nanoemulsions, liposomes, polymeric nanoparticles, solid lipid nanoparticles, cyclodextrin inclusion complexes, hydrogels, and targeted nanocarrier platforms. These formulations can enhance solubility, protect bioactive compounds from degradation, extend systemic circulation, increase tumour-specific accumulation and minimise off-target toxicity (134).

Despite the common perception that EOs are natural and therefore inherently safe, their safety profile requires comprehensive evaluation before clinical application. The biological activity of EOs is highly concentration-dependent, and several constituents may induce cytotoxic, hepatotoxic, nephrotoxic, neurotoxic, or genotoxic effects when administered at high doses or over prolonged periods. Additionally, allergic reactions, skin sensitisation, mucosal irritation, and oxidative tissue injury have been reported for specific EO components. A further critical yet often overlooked concern is the potential for herb-drug interactions. Numerous EO constituents can modulate cytochrome P450 enzymes, UDP-glucuronosyltransferases, ATP-binding cassette (ABC) transporters such as P-glycoprotein, and other drug-metabolising enzymes or transport systems. These interactions may alter the pharmacokinetics of concurrently administered chemotherapeutic agents, potentially reducing therapeutic efficacy or increasing systemic toxicity. Consequently, comprehensive toxicological assessments, pharmacodynamic and pharmacokinetic studies, dose optimisation, and interaction studies are essential before EOs can be safely integrated into standard oncology practice (132, 135).

Another limitation is the scarcity of robust preclinical and clinical evidence. Most studies investigating EO mediated targeting of CSCs are limited to two-dimensional cell culture systems or small animal models, which do not fully replicate the complexity of human tumours and their microenvironment. Preclinical investigations also vary considerably in EO composition, dosing regimens, extraction procedures, treatment duration, experimental models, and outcome measures, restricting direct comparison and reproducibility. Furthermore, only a small number of well-designed clinical trials have assessed the efficacy of EOs as anti-cancer agents. These trials often involve small patient cohorts, lack randomisation or appropriate controls and primarily evaluate supportive or palliative outcomes rather than direct antitumour activity. As a result, there is currently insufficient high-quality clinical evidence to recommend EOs as standalone anti-cancer therapies or as standard adjuncts to chemotherapy.

Future research should prioritise mechanistically driven translational studies that bridge the gap between laboratory discoveries and clinical application. Standardised EO formulations with well-defined chemical profiles should be assessed using advanced experimental models, including patient-derived organoids, three-dimensional spheroid cultures, patient-derived xenografts, and genetically engineered mouse models, which more accurately represent CSC biology and tumour heterogeneity. The integration of multi-omics technologies, such as genomics, transcriptomics, proteomics, metabolomics, and single-cell sequencing, will support the identification of molecular targets, predictive biomarkers, and patient populations most likely to benefit from EO-based therapies. Furthermore, systematic investigations into synergistic combinations of EOs with chemotherapy, radiotherapy, targeted therapies, or immunotherapy may provide effective strategies to eradicate CSCs, minimise toxicity, and overcome multidrug resistance (111).

## 9 Conclusion

CSCs represent a major obstacle to effective cancer therapy because of their capacities for self-renewal, phenotypic plasticity, tumour initiation, and intrinsic resistance to chemotherapy and radiotherapy. CSCs drive tumour recurrence, metastasis, and multidrug resistance through interconnected mechanisms, including activation of stemness-associated signalling pathways, epithelial–mesenchymal transition (EMT), enhanced DNA repair, metabolic adaptation, and interactions with the tumour microenvironment. As a result, CSCs are a critical therapeutic target. This review evaluates the emerging potential of EOs and their bioactive constituents as multitarget agents that modulate several CSC-associated pathways, such as Wnt/β-catenin, Notch, Hedgehog, PI3K/Akt/mTOR, NF-κB, and JAK/STAT. EOs have the potential to suppress CSC stemness, reverse drug resistance, induce apoptosis, and improve the efficacy of conventional therapies, offering a promising strategy to address a principal cause of treatment failure.

The principal scientific contribution of this review is the synthesis of current evidence indicating that EOs disrupt CSC biology and resistance mechanisms, rather than acting solely as conventional cytotoxic agents. This multitarget mode of action supports further investigation of EOs as adjuvant therapies that may sensitise resistant tumours and complement existing chemotherapy, targeted therapy, and immunotherapy.

Despite these promising findings, the clinical translation of EOs faces several significant challenges. The chemical heterogeneity of EOs, determined by plant species, geographical origin, cultivation conditions, extraction methods, and storage, compromises batch-to-batch reproducibility and highlights the need for standardised formulations and rigorous quality control. Furthermore, many EO constituents exhibit poor aqueous solubility, limited bioavailability, rapid metabolism, chemical instability, and short systemic half-lives, collectively limiting their therapeutic efficacy *in vivo*. Safety concerns also require thorough evaluation, as certain EO constituents may cause dose-dependent toxicity or interact with chemotherapeutic agents by modulating drug-metabolising enzymes and transporters. Additionally, the current evidence base consists mainly of *in vitro* and small-animal studies, whilst comprehensive pharmacokinetic investigations, long-term toxicological assessments, and well-designed randomised clinical trials remain lacking. These limitations currently preclude the routine clinical application of EO-based therapies.

Future research should focus on developing standardised, chemically characterised EO formulations, improving pharmacokinetic performance through advanced drug delivery systems such as nanocarriers, and validating efficacy using clinically relevant models, including patient-derived organoids and xenografts. The integration of multi-omics technologies and biomarker-guided approaches will further clarify EO-mediated CSC targeting and facilitate patient stratification. Importantly, adequately powered clinical trials are required to determine the safety, optimal dosing, pharmacokinetics, and therapeutic efficacy of EOs, particularly in combination with established anti-cancer therapies.

In summary, EOs represent a promising class of multitarget natural products with the potential to overcome CSC-mediated drug resistance and improve the durability of anti-cancer treatment. Although significant scientific and translational challenges remain, continued progress in standardisation, formulation technologies, mechanistic research, and clinical validation may enable the integration of EO-based therapies into future precision oncology strategies to prevent tumour recurrence, address therapeutic resistance, and improve long-term patient outcomes.
